# Probiotics for the treatment of depression and its comorbidities: A systemic review

**DOI:** 10.3389/fcimb.2023.1167116

**Published:** 2023-04-17

**Authors:** Jie Gao, Longyou Zhao, Yiwen Cheng, Wenhui Lei, Yu Wang, Xia Liu, Nengneng Zheng, Li Shao, Xulei Chen, Yilai Sun, Zongxin Ling, Weijie Xu

**Affiliations:** ^1^ Collaborative Innovation Center for Diagnosis and Treatment of Infectious Diseases, State Key Laboratory for Diagnosis and Treatment of Infectious Diseases, National Clinical Research Center for Infectious Diseases, The First Affiliated Hospital, School of Medicine, Zhejiang University, Hangzhou, Zhejiang, China; ^2^ Department of Laboratory Medicine, Lishui Second People’s Hospital, Lishui, Zhejiang, China; ^3^ Jinan Microecological Biomedicine Shandong Laboratory, Jinan, Shandong, China; ^4^ Department of Laboratory Medicine, Shandong Provincial Hospital Affiliated to Shandong First Medical University, Jinan, Shandong, China; ^5^ Department of Intensive Care Unit, The First Affiliated Hospital, School of Medicine, Zhejiang University, Hangzhou, Zhejiang, China; ^6^ Department of Obstetrics, The First Affiliated Hospital, School of Medicine, Zhejiang University, Hangzhou, Zhejiang, China; ^7^ School of Clinical Medicine, Institute of Hepatology and Metabolic Diseases, Hangzhou Normal University, The Affiliated Hospital of Hangzhou Normal University, Hangzhou, Zhejiang, China; ^8^ Department of Psychiatry, Lishui Second People’s Hospital, Lishui, Zhejiang, China

**Keywords:** comorbidity, depression, dysbiosis, microbiota-gut-brain axis, probiotics

## Abstract

Depression is one of the most common psychiatric conditions, characterized by significant and persistent depressed mood and diminished interest, and often coexists with various comorbidities. The underlying mechanism of depression remain elusive, evidenced by the lack of an appreciate therapy. Recent abundant clinical trials and animal studies support the new notion that the gut microbiota has emerged as a novel actor in the pathophysiology of depression, which partakes in bidirectional communication between the gut and the brain through the neuroendocrine, nervous, and immune signaling pathways, collectively known as the microbiota-gut-brain (MGB) axis. Alterations in the gut microbiota can trigger the changes in neurotransmitters, neuroinflammation, and behaviors. With the transition of human microbiome research from studying associations to investigating mechanistic causality, the MGB axis has emerged as a novel therapeutic target in depression and its comorbidities. These novel insights have fueled idea that targeting on the gut microbiota may open new windows for efficient treatment of depression and its comorbidities. Probiotics, live beneficial microorganisms, can be used to modulate gut dysbiosis into a new eubiosis and modify the occurrence and development of depression and its comorbidities. In present review, we summarize recent findings regarding the MGB axis in depression and discuss the potential therapeutic effects of probiotics on depression and its comorbidities.

## 1 Introduction

Depression is a psychiatric syndrome that characterized by slowed thinking, depressed mood, and reduced volitional activity as its main symptoms, and is often accompanied by suicidal tendencies and somatization symptoms, which pose a great threat to human health. Depression causes a huge economic burden on both families and society. Mounting evidence has shown a predominant increasing trend in the prevalence of depression in the general population (Weinberger et al., 2018), ranking 3^rd^ leading cause of global disability in healthy life years (Friedrich, 2017). Currently, depression become the 4^th^ most common illness in the world, with a prevalence rate of nearly 4.4% in both developed and developing countries (World Health Organization, 2017). According to the data from WHO, it is estimated that depression influences nearly 350 million people worldwide (Ledford, 2014), resulting in more than 800,000 suicide deaths annually. By 2030, depression is projected to rank as the first disease burden worldwide. Recently, the current COVID-19 pandemic has led to a great increase in depression, with an increase of nearly 53 million cases globally, 27.6% above the pre-pandemic levels (Salari et al., 2020). In China, depression is a major public health issue which has been reported to be the second leading cause of disability-adjusted life year (DALY) (Lu et al., 2021). According to the Blue Book on Depression in China (2022), the China Mental Health Survey reported that more than 95 million people suffering from depressive disorders account for 6.8% of the total population in China (Kessler and Bromet, 2013; Lu et al., 2021). Depression results in annual medical and social costs of up to 49.4 billion yuan, which has gradually become a major public health concern. Depression not only increases emotional suffering in patients but is also associated with an elevated prevalence of substantial present and future complications, such as irritable bowel syndrome (IBS), inflammatory bowel disease (IBD), heart disease, high cholesterol, obesity, diabetes mellitus (DM), and Alzheimer’s disease (AD), which will affect the quality of life (QoL) of these patients (Gold et al., 2020). Thus, there is an urgent need for scientists worldwide to address depression and its complications.

However, the pathogenesis of depression, which is important for its prevention and treatment, has not yet been clarified. Depression is caused by numerous environmental, genetic, and psychological factors. Currently, the major hypotheses for the development of depression include the monoamine reduction hypothesis, overactivation of the hypothalamus-pituitary-adrenal (HPA) axis, and the decrease in brain-derived neurotrophic factor (BDNF) levels (Sonali et al., 2022). All these hypotheses are closely related to the interaction of the gut-brain axis with the gut microbiota, named the microbiota-gut-brain (MGB) axis. In recent years, accumulating evidence has shown a close link between the gut and the brain, and that the gut microbiota can be involved in regulating brain development, anxiety, depression, cognitive function, and other central nervous system (CNS) activities (Adak and Khan, 2019; Ling et al., 2020; Simpson et al., 2021; Cheng Y. et al., 2022; Khan et al., 2022; Li and Chen, 2022; Li et al., 2022; Ling et al., 2022a; Ling et al., 2022b; Ling et al., 2022c; Ling ZX. et al., 2022). In patients with depression, neuronal apoptosis occurs in the frontal cortex, hippocampus, and amygdala, while the abnormalities of the metabolism, secretion, inter-synaptic transmission, and reuptake of monoamine transmitters such as norepinephrine, dopamine, and 5-hydroxytryptamine (5-HT) take place in the synaptic gap (Farooq et al., 2022; Suda and Matsuda, 2022; Zhu et al., 2022). Structural imaging techniques have revealed that the dorsolateral prefrontal cortex, superior parietal lobule hippocampus, and other parts of the hippocampus are reduced in volume (Doney et al., 2022). These responses are achieved through the immune, neuroendocrine, and vagal pathways of the MGB axis, which can be affected by the gut microbiota. In the immune pathway, the metabolites of the gut microbiota, such as short-chain fatty acids (SCFAs) and indole, can stimulate epithelial enterochromaffin cells (ECCs) to produce glucagon-like peptide-1 (GLP-1), which plays a vital role in reducing neuroinflammation (Cheng et al., 2019; Peirce and Alviña, 2019; Farooq et al., 2022; Sonali et al., 2022). In addition, gut dysbiosis-induced immune activation increases pro-inflammatory cytokines and reactive oxygen/nitrogen species levels, which leads to oxidative stress and causes hyperactivation of the HPA axis (Bravo et al., 2011; Simpson et al., 2021; Sonali et al., 2022). In the neuroendocrine pathway, various products of the gut microbiota can influence brain function, including neurotransmitters such as γ-aminobutyric acid (GABA), dopamine, serotonin, SCFAs, and tryptophan metabolites (Alli et al., 2022; Sonali et al., 2022). In the vagal pathway, the gut microbiota can modulate brain function through the vagus nerve. Vagal sensory neurons form various mechanosensory and chemosensory endings along the gastrointestinal tract that receive enterocephalic signals (Liu et al., 2021a; Décarie-Spain et al., 2023). In addition, ECCs can form synapses with adjacent nerves to assist the vagus nerve in receiving intestinal signals (Bravo et al., 2011; Sun et al., 2019; Snigdha et al., 2022). It is clear from the above that the gut microbiota and its metabolites regulate brain function through multiple pathways, which provides us with the novel idea of treating CNS diseases by modulating the gut microbiota.

## 2 Alterations of the gut microbiota in depression

Gut microbiota is a reservoir of trillions of bacteria, archaea, viruses, parasites, and fungi that live in the gut, which has been considered a forgotten organ of the human body. The gut microbiota plays a crucial role in a wide array of host processes, such as growth, development, physiology, immune regulation (Wastyk et al., 2021), intestinal mucosal barrier (Paone and Cani, 2020), nutrition (Valdes et al., 2018), colonization resistance (Ducarmon et al., 2019), and alterations in the gut microbiota are related to the development of various intestinal and extraintestinal diseases. During the past decades, scientists have made great efforts to explore how large-scale disruptions and dynamic shifts in the gut microbiota can drive phenotypic changes and disease states. With the strong evidence displayed by multi-directional evidence, recent findings confirm that the gut microbiota composition can affect brain development and behavior (Loughman et al., 2020; Carlson et al., 2021; Kelsey et al., 2021). Currently, the gut microbiota is no longer forgotten and has been called the “second brain” of the body (Ridaura and Belkaid, 2015). It is now well accepted that the gut microbiota is important for various brain processes such as neurogenesis, myelination, and microglial activation, which can also effectively regulate behavior and affect psychological processes such as mood and cognition (Dinan and Cryan, 2016). Gut dysbiosis can disturb the bidirectional crosstalk between the gut microbiota and the CNS, leading to numerous neurological conditions, including chronic pain, stress, anxiety, depression, autism spectrum disorder (ASD), AD, and Parkinson’s disease.

Recently, increasing evidence has shown a link between gut microbiota and depression *via* the MGB axis. In people with depression, alterations in gut microbiota patterns are evident, suggesting that the gut microbiota plays important roles in the pathogenesis and onset of depression (Jiang et al., 2015; Zheng et al., 2016; Ling et al., 2022a). The use of antibiotics can lead to gut dysbiosis, disrupt intestinal homeostasis, and increase the risk of depression (Hao et al., 2020). Changes in bacterial diversity and richness in patients with depression have been observed in several previous studies, although there was no consistent directional alteration in microbial diversity. Our previous study found that α-diversity increased significantly in adult patients with depression, whereas bacterial β-diversity did not change significantly (Jiang et al., 2015). However, the opposite patterns were found in childhood depression (Ling et al., 2022a). Previous studies have reported that several key functional bacteria at different taxonomic levels are associated with depression. Our previous study found that the decreased butyrate-producing bacteria (e.g., *Faecalibacterium*) were associated with the enrichment of intestinal pathogenic bacteria or opportunistic pathogens such as Enterobacteriaceae (Jiang et al., 2015). Interestingly, the key functional genus, *Faecalibacterium*, was correlated with the severity of depression symptoms and the difficulty in abstract thinking negatively (Valles-Colomer et al., 2019). Barandouzi et al. observed that depressed patients had a lower abundance of *Bacillus* spp., *Proteus* spp., *E. faecalis*, and *Sartorius* spp. and a higher abundance of *Actinomyces* and *Eggerthella* spp (Barandouzi et al., 2020).. These key functional bacteria were found to be correlated with the development of depression significantly, but their causal effects on depression remain unclear. Recently, animal models have emerged as valuable experimental tools for host-microbiome interaction research. Zheng et al. demonstrated that transferring the gut microbiota from depressed humans to germ-free (GF) mice could increase depression-like behavior in the recipient mice, supporting a causal relationship between gut microbiota and depression (Zheng et al., 2016). After successful antidepressant treatment, the depression-associated key functional differential bacteria decreased significantly compared to healthy controls, indicating that the depressed gut microbiota tended to reconstitute (Li N. et al., 2019; Farooq et al., 2022). Based on these clinical and preclinical findings, we tentatively inferred that maintaining or restoring the normal condition of the gut microbiota is associated with regression depression. These interactions are often shown to be produced by the immune, neuroendocrine, and vagal pathways with the gut microbiota and its metabolites *via* the MGB axis. Bacterial groups associated with gastrointestinal inflammation (e.g., Enterobacteriaceae, *Eggerthella*, and *Desulfovibrio*) were relatively abundant in patients with depression, and these patients had fewer anti-inflammatory SCFA-producing bacterial species, including butyrate-producing *E. faecalis* and *Clostridium* XIVa (Simpson et al., 2021). With the advent of multi-omics techniques, such as metagenomics, metabolomics, proteomics, and culturomics, depression-associated key functional bacteria can be identified into species or strain levels, which allows us to clarify the specific functions of the depressed bacteria and screen these species as biomarkers for the intervention and treatment of depression and related complications.

Traditionally, depression treatments target the brain with psychotherapy and/or different drugs, such as 5-HT reuptake inhibitors (SSRI), 5-HT, and norepinephrine reuptake inhibitors (SNRI). However, the prevalence and burden of depression remained unchanged (Malhi and Mann, 2018). Recent advances have observed that these conventional treatments not only regulate the brain directly (Cipriani et al., 2018), but also affect the gut microbiota (Davey et al., 2013). Correcting abnormal gut microbiota could alleviate depression, suggesting that targeting on the gut microbiota could be considered as a promising and tractable therapy for depression. The modulation of the gut microbiota has been highlighted in the treatment of mental disorders, including depression and its comorbidities. To explore more potential possibilities for the treatment of depression, this review explored the use of probiotics in depression and for diseases in which depression is a co-morbidity. Here, we describe the pathogenesis associated with the MGB axis in depression and discuss the potential therapeutic effects of probiotics on depression and its comorbidities, with the expectation that probiotics will become a new and effective treatment for depression and its comorbidities in the future.

## 3 Underlying mechanisms of the gut microbiota in depression

**Figure 1:**
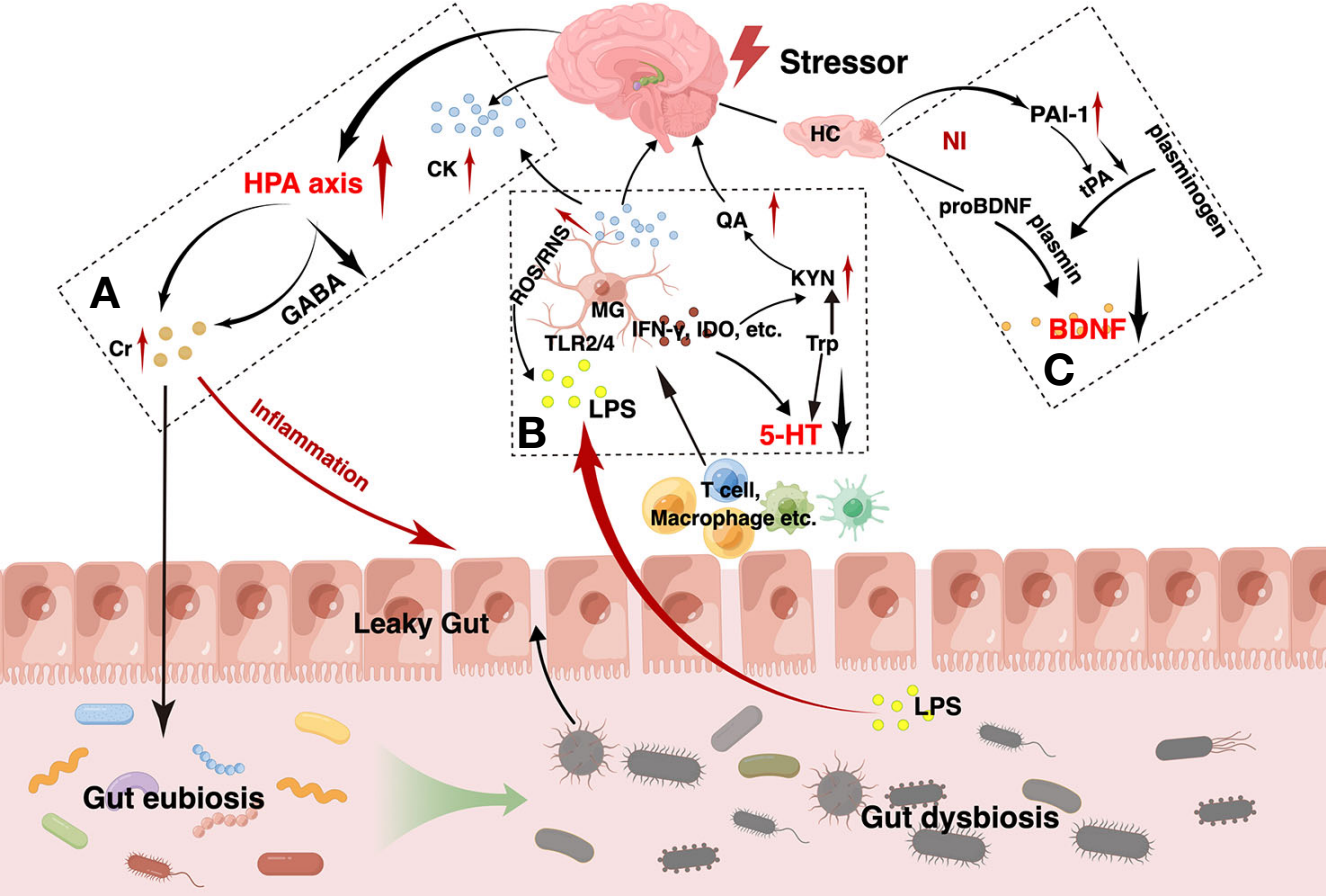
The main pathogenesis hypotheses for depression via the gut-brain axis. As shown, the hypothalamus-pituitary-adrenal (HPA) axis of the brain is activated by various stressors such as psychological stress and the increased pro-inflammatory cytokines **(A)**. Due to the disruption of the g-aminobutyric acid (GABA) negative feedback in depression, however, the HPA axis abnormally activated, leading to persistent elevation of cortisol. High level of cortisol contributes to peripheral inflammation and can disrupt the normal gut microbiota. Gut dysbiosis, inflammation and other factors in combination lead to impaired intestinal barrier function, called leaky gut, which manifests as increased intestinal permeability, decreased intestinal villi length and colonic crypts depth. Intestinal immune cells such as T cells, macrophages, and so on, and gram-negative bacteria-produced lipopolysaccharide (LPS) in the gut can translocate from gut into brain, which can act on the microglia and lead to neuroinflammation. In turn, the over-activated microglia can release amount of indoleamine 2,3-dioxygenase (IDO), interferon γ (IFN-γ) and so on, resulting in a decrease in tryptophan metabolism to 5-hydroxytryptamine (5-HT) and an increase in the neurotoxic kynurenine, quinolinic acid **(B)**. Meanwhile, neuroinflammation can reduce the level of brain-derived neurotrophic factor (BDNF) that is produced in the atrophied hippocampus and prefrontal cortex. Abnormally reduced plasminogen activator inhibitor-1 (PAI-1) is also considered as a possible causative agent **(C)**. All these factors interact with each other and contribute to the onset and worsening of depression.

There is growing evidence to support the role of the gut microbiota in regulating host behavior and brain function. Although the exact mechanisms by which the gut microbiota causes or alters depression are not fully understood, the vast evidence from previous clinical and preclinical studies supports the hypothesis that the gut microbiota can affect the development of depression, mainly through the HPA axis, inflammation, and modify the abundance of BDNF. Clarifying the underlying mechanisms between gut microbiota and depression can contribute to the effective prevention and treatment of depression (**Figure 1**).

### 3.1 HPA axis

The HPA axis is a feedback pathway consisting of the hypothalamus, pituitary, and adrenal glands and is involved in the control of the stress response. The HPA axis is an important component of the MGB interaction (Cryan and O'mahony, 2011; Frankiensztajn et al., 2020). Under normal conditions, environmental, emotional, and physiological stressors increase the systemic levels of pro-inflammatory cytokines, which in turn secrete corticotropin-releasing hormone (CRH) by triggering secretion from the paraventricular nucleus of the hypothalamus. Increased CRH levels activate adrenocorticotropic hormone (ACTH) secretion from the anterior pituitary. ACTH subsequently results in the release of glucocorticoids from the adrenal cortex. When glucocorticoid levels reach their peak, negative feedback regulation is triggered *via* the GABA pathway, resulting in a decrease in cortisol secretion. This constitutes a complete HPA axis pathway, which ensures the stability of cortisol levels *in vivo* (Suda and Matsuda, 2022). However, this mechanism is disrupted in patients with depression. As we know before, the HPA axis is negatively regulated through the GABA pathway, which proceeds by increasing glutamate reuptake by presynaptic neurons when cortisol levels peak, and within the neuron, glutamate is converted to GABA as a precursor, followed by GABA exiting the neuron into the synaptic vesicles, crossing the synaptic gap and binding to GABA receptors on the postsynaptic membrane, thus establishing an aminobutyric acidergic synapse (Herman et al., 2004; Cullinan et al., 2008; Sarkar et al., 2011; Colmers and Bains, 2018; Duman et al., 2019). GABA acts as an inhibitory neurotransmitter that inhibits CRF neurons, ultimately reducing the secretion of cortisol (Giordano et al., 2006). That is, the GABA content is elevated during normal action. However, the dexamethasone suppression tests observed lesser levels of GABA levels in depressed patients which are unable to regulate excessive cortisol levels (Carroll et al., 1981), while excessive cortisol levels can lead to gut dysbiosis eventually (Łaniewski and Herbst-Kralovetz, 2022; Couch et al., 2023). Gut dysbiosis promotes the growth of gram-negative bacteria, releases increased immunogenic lipopolysaccharide (LPS), disrupts intestinal permeability, leads to leaky gut and endotoxemia, and causes translocation of bacterial components in the gut lumen due to the inflammatory response. These inflammatory mediators in turn stimulate the HPA axis, exacerbating its hyperactivation and promoting neuroinflammation, leading to depressive behavior (Zheng et al., 2016; Liu et al., 2021b; Sonali et al., 2022).

As mentioned above, high cortisol levels lead to a compromised intestinal mucosal barrier, which allows T cells, giant cells, and antigens to flow into the brain, activate microglia, and trigger inflammation (Doney et al., 2022). It can also lead to inflammation through the binding of effluent LPS to toll-like receptor (TLR) on microglia (Qin et al., 2007). Indeed, excessive microglial activation is the cornerstone of neuroinflammation. Multiple factors can lead to its overactivation to produce neuroinflammation and eventually lead to depression.

### 3.2 Inflammation

Inflammation is a key causative factor in the development of depression. However, the exact mechanisms underlying inflammation-related depression are still elusive. Typically, over-activation of the HPA axis by stress can cause abnormally high cortisol levels and disruption of the intestinal barrier, allowing the outflow of gut microbiota-derived endotoxins into circulation. This causes increased pro-inflammatory cytokines and decreased anti-inflammatory cytokines, leading to peripheral inflammation, which is associated with depressive symptoms. Some studies have reported increased interleukin-1β (IL-1β) and interleukin-6 (IL-6) and decreased IL-4 and IL-10 in patients with depression (Berk et al., 2013; Wong et al., 2016; Ling et al., 2022a). Then, pro-inflammatory factors cross the blood-brain barrier (BBB) into the brain through blood circulation, activating microglia, releasing reactive nitrogen and oxygen, and damaging brain epithelial cells leading to neuroinflammation, which can lead to mental illness (Peirce and Alviña, 2019; Doney et al., 2022). At the same time, indoleamine 2,3-dioxygenase (IDO) is a key enzyme in inflammation-induced depression. The pro-inflammatory factors also activate IDO enzyme activity, which causes tryptophan to be broken down into kynurenine rather than 5-HT, reducing 5-HT concentrations (Peirce and Alviña, 2019; Walker et al., 2019). Furthermore, pro-inflammatory factors disrupt tetrahydrobiopterin, an essential factor for monoamine synthesis, leading to impaired synthesis of 5-HT, dopamine, and other neurotransmitters (Dantzer et al., 2008; Miller and Raison, 2016).

As mentioned earlier, fecal microbiota transplantation (FMT) from depressed patients to GF mice causes morbidity in the mice. Compared with normal mice, GF mice are immunocompromised and lack regulatory T cells (Tregs) (Zheng et al., 2016). Thus, these findings suggest a mediating role of the gut microbiota in immune response and depression. Mason et al. found that anti-inflammatory bacterial groups such as *Bacteroides* and the *Clostridium leptum* subgroup are reduced in those diagnosed with depression (Mason et al., 2020), suggesting that gut dysbiosis, especially the decrease of these anti-inflammatory regulatory bacteria, plays an important role in the pathophysiology of depression. Given the key role of inflammation in depression, strong evidence has supported the novel concept of the gut microbiota-inflammation-brain axis, in which the gut microbiota can alter brain function through inflammatory signaling pathways and affect depression-like behavior. Thus, targeting the gut microbiota to modulate the inflammatory response in individuals with depression may represent a useful therapeutic approach for depression.

### 3.3 Decreased level of BDNF

BDNF is a neurotrophin that can regulate the growth and plasticity of neurons and synapses (Bercik et al., 2011a). It is distributed in the hippocampus widely and has been considered as a key transducer of antidepressant effects. Generally, BDNF is first synthesized a precursor protein, pro-brain-derived neurotrophic factor (pro-BDNF), which is further processed into the mature form by fibrinolytic enzymes. BDNF can increase synaptic plasticity, promote neurogenesis, especially in the hippocampus, and maintain and promote the developmental differentiation and regeneration of various neurons, especially pentraxin and dopaminergic neurons, whereas pro-BDNF induces neuronal death and synaptic pruning (Bai et al., 2016). Fibrinolytic enzymes are converted from fibrinogen in the presence of tissue plasminogen activator, which can be inhibited by plasminogen activator inhibitor-1 (PAI-1), leading to the accumulation of pro-BDNF. Previous studies found that PAI-1 increases in the prefrontal cortex and hippocampus of chronically stressed rats (Party et al., 2019; Zhang W. et al., 2022). The decreased BDNF can be found in patients with depression (Jiang et al., 2015; Youssef et al., 2018), whereas antidepressant treatment can increase the levels of BDNF (Martinotti et al., 2016). Kuhlmann et al. observed that the levels of BDNF were negatively correlated with the severity of depression (Kuhlmann et al., 2017). Serum BDNF levels correlate with hippocampal volume, and insufficient BDNF levels can impair neurogenesis and lead to the onset of depression (Erickson et al., 2012; Von Bohlen Und Halbach and Von Bohlen Und Halbach, 2018). These findings suggest the possibility of using serum BDNF level as an indicator of disease activity and treatment response. Evidence from animal studies using GF, antibiotic-treated, depression models, and FMT mice has also demonstrated lower levels of BDNF in the hippocampus and cortex than in healthy controls (Clarke et al., 2013; Jang et al., 2021; Suda and Matsuda, 2022). Gut eubiosis can increase the activation of cAMP response element binding (CREB) in the hippocampus, prefrontal cortex, and amygdala, leading to an increase in BDNF production (Gerhard et al., 2016; Methiwala et al., 2021). In addition, the gut microbiota can modulate the conversion of pro-BDNF into BDNF. Administration of microbiota products to mice increases BDNF levels in the hippocampus (Carabotti et al., 2015; Maqsood and Stone, 2016). Previous interventional studies have revealed that probiotics can restore the levels of pro-BDNF and BDNF in various brain regions which are related to the development of depression-like behavioral phenotypes (Sun et al., 2018; Mohammed et al., 2020). These findings suggest that re-establishment of the gut microbiota may contribute to the increased levels of brain BDNF and modulate host behavior.

The aforementioned mechanisms provide an introduction to the pathogenesis of depression briefly. In fact, the causes of depression are inconclusive and there are numerous hypotheses about its pathogenesis, of which the above-mentioned are few and incomplete. The premise we are dealing with is related to the gut microbiota and can be treated with probiotics and other related mechanisms. Based on the above mechanisms, we will explore the existing targets that can act through probiotic therapy and explore the possible applications of probiotics in the future, which often act through the MGB axis interaction, reflecting the great role and potential of the gut microbiota in regulating neurological diseases.

## 4 Therapeutic potential of probiotics on depression

Various antidepressant agents are available to treat depression, including monoamine oxidase inhibitors, tricyclic antidepressants, selective SSRI, nonselective SNRI, selective norepinephrine reuptake inhibitors, and other miscellaneous agents such as mirtazapine (Keller et al., 2002; Cryan and Dinan, 2015). However, nearly 30% of patients with depression are resistant to any treatment. Thus, novel antidepressant agents and strategies are required (Miyanishi and Nitta, 2021). Recent microbiota studies have demonstrated a strong link between depression and the gut microbiota. Miyaoka et al. has observed that the combination of antidepressants and probiotics is more effective to treat drug-resistant depression (Miyaoka et al., 2018). Preclinical studies and clinical trials suggest that modifying the composition of the gut microbiota *via* probiotic supplementation have been proven to be beneficial in treating or preventing human diseases (Zhao et al., 2018; Sanders et al., 2019; Edwards et al., 2020; Sun et al., 2021), which may be a viable adjuvant treatment option for patients with depression.

**Figure 2:**
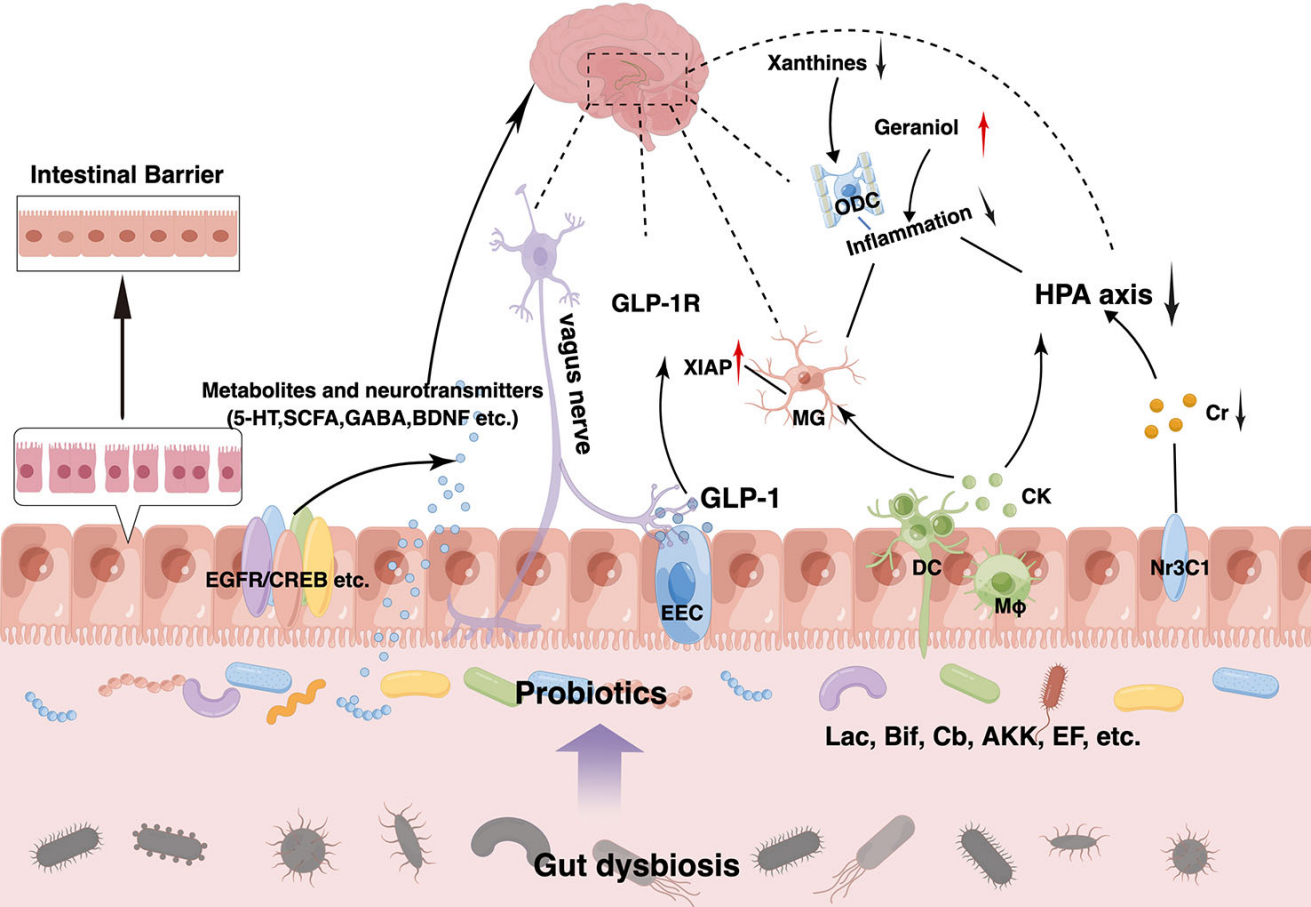
Therapeutic effects of probiotics on depression and its comorbidities. Probiotics exert antidepressant effects at multiple targets. Supplementation of probiotics can increase beneficial microorganisms and reduce harmful ones to achieve new gut eubiosis. Meanwhile, they can produce beneficial substances such as 5-hydroxytryptamine (5-HT), short-chain fatty acids (SCFAs) and brain-derived neurotrophic factor (BDNF) and so on by themselves or indirectly through upregulation of beneficial microbiota, which can act on various receptors on the intestinal epithelium such as EGFR, CREB, Nr3C1, etc. Moreover, probiotics can enhance intestinal barrier function in a variety of ways, such as upregulating mucus production by goblet cells, enhancing zonula occludens-1 (ZO-1), occludin, and claudin-1 expression, and reducing inflammation. Probiotics can also regulate brain function and improve neurological function through the vagus nerve, glucagon-like peptide-1 (GLP-1) pathway, etc. And it can activate the immune system to produce anti-inflammatory factors, alleviating circulatory inflammation and neuroinflammation while downregulating the hyperactive microglia and hypothalamus-pituitary-adrenal (HPA) axis. Similarly, decreased xanthines levels and elevated geraniol levels can also reduce inflammation.

Probiotics are live microorganisms that, when administered in adequate amounts, confer a health benefit to the host by changing the composition of the host’s microbiota in a certain area (Hill et al., 2014). They play a vital role in maintaining a healthy gut by regulating the host mucosa and systemic immune function or by regulating the balance of microbiota in the gut (Sánchez et al., 2017; Clemente et al., 2018). Probiotics can tolerate stomach acid and bile salts, adhere to host intestinal epithelial cells, and remove or reduce the adhesion of pathogenic bacteria (Selle and Klaenhammer, 2013; Ducarmon et al., 2019; Mazloom et al., 2019). Owing to their non-toxic side effects and high stability, probiotics are increasingly used in the prevention and treatment of intestinal disorders such as IBS, IBD, antibiotic-associated diarrhea, and other disorders (Park et al., 2018; Glassner et al., 2020). Gut microbiota modulation with probiotics has become a hot topic in the treatment of mental disorders, including depression, although it is still in its infancy stage. Probiotics acting *via* the MGB axis can influence brain development, function, and behavior (Desbonnet et al., 2014; Buffington et al., 2016). This has prompted growing interest in the possibility of targeting the gut microbiota to beneficially impact depression. The concept of “psychobiotics”, proposed by Dinan et al., emphasizes the potential of probiotics in mental disorders treatment (Dinan et al., 2013). Psychobiotics can convey benefits to the host’s mental health *via* dynamic MGB crosstalk. An emerging body of evidence suggests possible antidepressant effects resulting from probiotic supplementation, which can normalize depression-associated physiological outputs, such as corticosterone, noradrenaline, BDNF, and immune function. The promising role of probiotics in depression *via in vivo* and *in vitro* studies have laid a strong foundation for clinical application. Several recent randomized controlled trials (RCTs) have demonstrated that probiotics can alleviate depressive symptoms in participants both with and without a clinical diagnosis of depression effectively (Goh et al., 2019; Amirani et al., 2020; Chao et al., 2020; Dehghani et al., 2022). Probiotics exhibit antidepressant properties in the absence of other therapeutic options (Nikolova et al., 2021). Thus, microbiota-based interventions with probiotics may possess greater therapeutic potential for depression treatment, which can be used as an adjunct to current approaches (**Table 1**). Common probiotic strains, such as *Lactobacillus* spp., *Bifidobacterium* spp., *Akkermansia* spp., *Clostridium* spp., and *Enterococcus* spp., have been used to treat depression in clinical and animal studies, either as a single agent or in combination with other potential psychobiotics (**Figure 2**). However, it is important to note that these benefits are strain-specific. We selected some strains that have already played an effective role in the treatment of depression to illustrate the specific mechanism of its action, clarify its dosage, periodicity, and other key information in the current treatment regimen, and pave the way for the further role of probiotics, which are expected to become new options for the treatment of depression (**Table 2**).

**Table 1:** Clinical evaluation of probiotics on depression and its comorbidities.

Strains	Study design	Population characteristics	Intervention	Control/placebo group	Duration	Clinical findings	References
LGG HN001	RCT	380 women (14-16 weeks gestation) Probiotics (n=193), Placebo (n=187)	HN001 (6×10^9^ CFU daily)	Corn-derived maltodextrin	6m	Lower depression scores: HN001 mean = 7.7 (SD=5.4), placebo 9.0 (SD=6.0), p=0.037	(Slykerman et al., 2017)
*L. plantarum* PS128	RCT	40 participants with self-reported insomnia PS128 group (n=21), Placebo (n=19)	PS128 (6×10^10^ CFU) daily	microcrystalline cellulose (6×10^10^ CFU) daily	30d	↓BDI-II scores	(Ho et al., 2021)
*L. plantarum* PS128	A Preliminary open trial	11 patients with MDD	Capsule contained 3×10^10^ CFU of PS128 twice a day	/	8w	↓HAMD-17 scores; ↓DSSS scores	(Chen HM. et al., 2021)
*L. plantarum* 299v	RCT	60 patients with MDD LP299v group (n = 30), Placebo (n = 30)	capsule contained 10×10^9^ CFU of LP299v twice a day	crystalline cellulose powder	8w	Improvement in APT and in CVLT total recall of trials 1–5; ↓KYN; ↑3HKYN : KYN	(Rudzki et al., 2019)
*L. helveticus* and *B. longum*	three-arm parallel design, RCT	81 patients with MDD Probiotics (n=28), Prebiotics (n=27), Placebo (n=26)	10×10^9^ CFU per 5 g sachet/day	excipients	8w	↓BDI score; ↓kynurenine/tryptophan; ↑tryptophan/isoleucine	(Kazemi et al., 2019)
*L. paracasei Shirota*	RCT	69 patients with depression LcS group (n=38), Placebo (n=31)	100 mL beverage of LcS (10^10^CFU)	the same fermented dairy beverage without any bacteria	9w	↑*Adlercreutzia*, *Megasphaera* and *Veillonella*; ↓Rikenellaceae_RC9_gut_group, *Sutterella* and *Oscillibacter*; ↓IL-6; Relieve constipation	(Zhang et al., 2021)
*L. paracasei Shirota*	A single-arm trial	15 patients with MDD and 3 patients with BD	2 bottles of fermented milk containing at least 4.0×10^10^ CFU of LcS per bottle (80 mL) per day	/	12w	↓HAMD21	(Otaka et al., 2021)
*B.longum* NCC3001	RCT	44 adults with IBS and diarrhea or a mixed-stool pattern and mild to moderate anxiety and/or depression BL group (n= 22), Placebo (n = 22)	BL (10^10^ CFU/1g)	maltodextrin	6w	↓HAD-D scores; ↑Quality of life score; ↓Responses to negative emotional stimuli; ↓Urine levels of methylamines and aromatic amino acids metabolites	(Pinto-Sanchez et al., 2017)
*B.breve* CCFM1025	RCT	45 patients with MDD patients (n = 45) CCFM1025 group (n = 20), Placebo (n = 25)	CCFM1025 (10^10^ CFU) daily	maltodextrin	4w	↓HDRS-24 scores; ↓MADRS scores; ↓BPRS scores; ↓GSRS scores; ↓Serum serotonin turnover	(Tian et al., 2022)
*B.bifidum* BGN4 *and B.longum* BORI	RCT	63 healthy elders (≥65 years) Probiotics (n=32), Placebo (n=31)	Two capsules twice per day (1×10^9^ CFU of BGN4 and BORI)	soybean oil	12w	↓*Eubacterium*, *Allisonella*, Clostridiales, and Prevotellaceae; ↑BDNF; ↓Mental flexibility score; ↓Stress score	(Kim CS. et al., 2021)
Mixture (*B.bifidum* W23*,B.lactis* W51*, B. lactis* W52*, L. acidophilus* W37, *L. brevis* W63*, L. casei* W56*, L. salivarius* W24*, L.lactis* W19 *and L.lactis* W58)	RCT	71 participants with depressive symptoms Probiotics (n = 34), Placebo (n = 37)	Two 2g sachets of mixture for each day (total cell count 1 × 10^10^ CFU/day	two 2g sachets daily of the freezedried maize-starch and maltodextrins	8w	Reduction in cognitive reactivity	(Chahwan et al., 2019)
*Streptococcus thermophilus* NCIMB 30438*, B.breve* NCIMB 30441*, B.longum* NCIMB 30435, *B.infantis* NCIMB 30436*, L.acidophilus* NCIMB 30442*, L.plantarum* NCIMB 30437, *L.paracasei* NCIMB 30439*, L.delbrueckii subsp.* *Bulgaricus* NCIMB 30440	RCT	47 patients with current depressive episodes Probiotics (n=21), Placebo (n= 26)	Probiotic mixture (900 billion CFU/day)	maltose	4w	↓HAM-D scores; ↑*Lactobacillus;* ↑Gray matter volume in calcarine sulcus; Alter putamen’s activation during emotion processing	(Schaub et al., 2022)
probiotic NVP-1704, a mixture of *L. reuteri* NK33 and *B. adolescentis* NK98	RCT	156 healthy adults with subclinical symptoms NVP-1704 group (n = 78), Placebo (n = 78)	NVP-1704 (2.0 × 10^9^ CFU for NK33 and 0.5 × 10^9^CFU for NK98) daily	maltodextrin	8w	Reduction in depressive symptoms Improvement in sleep quality; ↓IL-6; ↑Bifidobacteriaceae; ↑Lactobacillacea; ↓Enterobacteriaceae	(Lee et al., 2021)
Mixture (containing *L. fermentum* LF16, *L. rhamnosus* LR06, *L. plantarum* LP01, and *B. longum* BL04)	RCT	38 healthy volunteers Probiotics group (n=19), Placebo group (n=19)	probiotic mixture (4 × 10^9^ CFU/AFU) daily	maltodextrin	6w	Improvement in mood; Reduction in depressive mood state, anger, and fatigue; Improvement in sleep quality	(Marotta et al., 2019)

3HKYN, 3-hydroxykynurenine; AFU, active fluorescent unit; AhR, aryl hydrogen receptor; APT, Attention and Perceptivity Test; B. adolescentis, Bifidobacterium adolescentis; B. bifidum, Bifidobacterium bifidum; B. breve, Bifidobacterium breve; B. infantis, Bifidobacterium infantis; B. lactis, Bifidobacterium lactis; B. longum, Bifidobacterium longum; BAI, Beck Anxiety Inventory; BD, Bipolar Disorder; BDI, Beck Depression Inventory; BDI-II, Beck Depression Inventory-II; BDNF, Brain-derived neurotrophic factor; CERAD-K, The Korean version of the Consortium to Establish a Registry for Alzheimer’s Disease; CFU, colony forming units; CRS, chronic restraint stress; CVLT, California Verbal Learning Test Total; DSSS, Depression and Somatic symptoms Scale; EPM, the elevated plus-maze; FST, the forced swimming test; GPX2, glutathione reductase 2; GSRS, Gastrointestinal Symptom Rating Scale; HAD-D, HAD-depression; HAMD-17, Hamilton Depression Rating Scale-17 items; HAMD-21, Hamilton Depression Rating Scale-21 items; HDRS-24, Hamilton Depression Rating scale-24 Items; IBS, Irritable Bowel Syndrome; IL-1β, interleukin-1β; IL-6, interleukin 6; IL-8, interleukin 8; ILA, indole-3-lactic acid; KYN, kynurenine; L. acidophilus, Lactobacillus acidophilus; L. brevis, Lactobacillus brevis; L. casei, Lactobacillus casei; L. delbrueckii, Lactobacillus delbrueckii; L. fermentum, Lactobacillus fermentum; L. helveticus, Lactobacillus helveticus; L. paracasei, Lactobacillus paracasei; L. plantarum, Lactobacillus plantarum; L. salivarius, Lactobacillus sailvarius; LGG HN001, Lactobacillus rhamnosus GG HN001; MADRS, Montgomery-Asberg Depression Rating Scale; MDD, major depression; NF-κB, nuclear factor kappa B; NQO1, NAD(P) H dehydrogenase; Nrf2, nuclear factor erythroid 2-related factor 2; RCT, randomized controlled trial; SOD2, superoxide dismutase 2; STAI1, State-Trait Anxiety Inventory 1; TNF-α, tumor necrosis factor alpha.

↓: downregulation; ↑: upregulation.

**Table 2:** Animal studies of probiotics on depression and its comorbidities.

Strains	Animals	Intervention	Control/placebo group	Duration	Clinical findings	References
*L. rhamnosus* JB-1	Adult male BALB/c mice (n = 36) JB-1 group (n = 16), Control group (n = 16)	JB-1 (10^9^CFU) daily	Broth without bacteria	28d	Induced region-dependent alterations in GABA mRNA in the brain; ↓Corticosterone; ↓Depression-related behavior	(Bravo et al., 2011)
*L. rhamnosus* JB-1	48 male Wistar rats Control group (n= 10), JB-1 group (n= 19), Placebo (n = 19))	JB-1(3.4 × 10^8^ CFU) daily by oral gavage	PBS	4w	↑GSH; ↑Glutamate; ↑tNAA	(Kochalska et al., 2020)
*L. rhamnosus* JB-1 *and L. reuteri* 6475	15 male BALB/c mice JB-1 group (n=5), *L.reuteri* 6475 (n=5), Placebo (n=5)	orally gavaged with JB-1 (2 × 10^9^) or *L. reuteri* 6475 (2 × 10^9^) daily	PBS	2w	Modulated the activity of interferon signaling, JAK/STAT, and TNF-α *via* NF-κB pathways; ↑FKBP1A, SNRPN, CELF4, GPM6B, APBB1, NCDN, RUNDC3A, CPE, CLSTN1	(Haas-Neill et al., 2022)
*B. longum* and *L. rhamnosus*	50 male Wistar rats 5 groups (n=10) each Control group, CUMS group, FOS/GOS group, BL group, Lr group	BL/Lr (1 × 10^10^ CFU)	saline	28d	Ameliorate the CUMS‐induced loss of weight and depressive‐like behaviors; ↓Colonic 5‐HT; ↑Central 5‐HT; Modulate gut microbiota disturbed by CUMS	(Li H. et al., 2019)
*L. rhamnosus* zz-1	60 male C57BL/6 mice, 5 groups (n = 12) each CUMS group, L.Rzz 9 group, L.Rzz 8 group, L.Rzz 7 group, Control group	zz-1 (2 × 10^9/^10^8^/10^7^ CFU)	saline	6w	↑Body growth rate; ↑Sucrose preference; ↓Immobility time; ↑Curiosity and mobility; ↓ACTH, CORT; ↑CRF; ↑5-HT, NE, and DA; ↑BDNF and TrkB; ↓Intestinal damage; ↓Intestinal inflammation; *↑*Firmicutes; ↑Firmicutes/Bacteroidetes; ↑Lachnospiraceae NK4A136 group; *↓*Bacteroidetes, *Bacteroides*, and *Muribaculum*	(Xu J. et al., 2022)
*L. reuteri* NK33 and *B. adolescentis* NK98	42 male C57BL/6 mice 6 groups (n=7) each (NC, C, PC, NK33, NK98, and Mix)	NK33 (1 × 10^9^ CFU) NK98 (1 × 10^9^ CFU) Mix (1 × 10^9^ CFU 1:1 mixture of NK33 and NK98)	maltose buspirone	5d	↓LPS; ↓GC; ↓IL-6; ↑BDNF; ↓NF-κB activation; ↓Proteobacteria; ↓Suppress anxiety/depression; Activated microglia infiltration; Suppress IS-induced colon shortening, myeloperoxidase activity	(Jang et al., 2019)
*L. delbrueckii*	32 C57BL/6J mice 4 groups (n=8) each normal control group, control + Lac group, model group, model + Lac group	10^9^ CFU of Lac daily by oral gavage	saline	7d	↑ZO-1 and E-cadherin; ↓Overactivation of microglia; ↑DAT; ↓IL-1β, TLR4, and NLRP3; Inhibited dysbiosis	(Qiu et al., 2021)
*L. casei*	20 male Sprague-Dawley rats 4 groups (n=5) each CUMS+Lac, CUMS+paroxetine, CUMS+saline, Saline	*L. casei* (8 × 10^8^ CFU)	saline	4w	↑Body growth rate; ↑Sucrose preference; ↓Immobility time; ↑Moving distance and velocity; Amend the gut microbiota structure changes; ↑NE, DA, 5-HT, BDNF, TrkB, NR1; ↓Activations of ERK1/2 and p38 MAPK signal pathways	(Gu et al., 2020)
*L. helveticus* and *B. longum*	47 male Syrian hamsters divided into 3 groups placebo group(n=7), low dose group(n=20), high dose group(n=20)	Low dose:10^9^ CFU daily High dose:10^10^ CFU daily	excipients	14d	↑IL-4, IL-5, and IL-10; ↑Social avoidance; ↓Social interaction; ↓Microbial richness	(Partrick et al., 2021)
*L. paracasei* HT6	24 Wistar rats 4 groups (n=6) each control + Pro, MS + Pro, M+P+S+Pro, MS+S−M + Pro	10^9^ CFU as the fixed dose (starting from 20 µl on PND-2 to 100 µl on PND-16 with an incremental increase in volume of 20 µl for every 3 days)	/	15d	Beneficial effect on anxiety-like behavior; Normalize levels of ACTH, CORT, GR, 5-HT, DA, NA; Prevent stress-associated GluR1-GR altered interactions	(Karen et al., 2021)
*B. dentium*	Adult germ-free mice germ-free (MRS broth): males=27, females=30, live *B.dentium*: males=28, females=29, live *B.ovatus*: males=5, females=5, heat-killed *B.dentium*: males =5, females=5.	oral-gavaged with 6.4 × 10^7^ CFU of live *B dentium*, 6 × 10^7^ CFU of live *B ovatus*, 6.4 × 10^7^ CFU of heat-killed *B dentium*	sterile MRS broth	2w	↑5-HT; ↑Fecal acetate; ↑5-HT receptors 2a, 4, and serotonin transporter	(Engevik et al., 2021)
*B. dentium*	74 adult Swiss Webster germ-free mice *B.dentium* group (n=33), heat-killed *B.dentium* (n=10), control group (n=31)	oral gavage with 3.2×10^8^ CFU *B.dentium*	sterile MRS broth	2w	↑Goblet cell markers, Klf4, Tff3, Muc2, Relm-β; ↑Glycosyltransferases; ↑Acidic mucin-filled goblet cells; ↑Autophagy mediated calcium signaling	(Engevik et al., 2019)
*B. dentium*	90 male C57BL/6J mice randomly divided into 11 groups including control group and *B.dentium* N8 group	*B.dentium* N8 (1×10^9^ CFU)	sterile silk milk	21d	↑Adhesion ability; ↓*Escherichia coli* ATCC 25922 adhesion to HT-29 cells; ↑TEER; ↓Paracellular permeability of Caco-2 cells; ↑ZO-1, occludin, and claudin-1 mRNA expression; ↓TLR4 and pro-inflammatory cytokines (TNF-α, IL-1β, IL-6) mRNA expression	(Zhao et al., 2021)
*B. adolescentis*	Male ICR mice Exp1:5 groups (n=10) Con group, Ami group, Bif 0.25 group, Bif 0.5 group, Bif 1 group Exp2:3groups (n=12) Con group, CRS group, Bif+CRS group	Exp1:0.25/0.5/1.0×10^9^ CFU B. *adolescentis* Exp2:0.25×10^9^ CFU B. *adolescentis*	Exp1:10 mL/kg distilled water Exp2:10 mL/kg distilled water	21d	↑The time spent in the center of the OFT apparatus, the percentage of entries into the open arms of the EPM and the percentage of time spent in the open arms of the EPM; ↓Immobility duration in the tail suspension test and the FST; ↑*Lactobacillus*; ↓*Bacteroides*; ↓IL-1β, TNF-α, NF-κB p65, Iba1; ↑BDNF	(Guo et al., 2019)
*L. plantarum 90sk and B. adolescentis*	48Male BALB/c mice 4 groups (n=12) each Mixture of strains group, Fluoxetine group (10 mg/kg), Monosodium glutamate group 2.5 mg (100 mg/kg), Distilled water group	10^8^ CFU LP90sk and 10^7^ CFU *B. adolescentis* 150 daily	/	14d	Decrease the duration of immobility of mice in FST	(Yunes et al., 2020)
*B. breve* CCFM1025	40 chronically stressed male adult C57BL/6 mice, 4 groups (n=10) each control, CUMS, Fluoxetine, CCFM1025	CCFM1025 of 0.1 ml/10g body weight, 10^9^ CFU/mL daily.	vehicle	5w	Alleviate the hyperactive HPA axis and inflammation; Restore the abnormal gut microbiota; ↓the pCREB-c-Fos Pathway; ↑BDNF; ↑SCFAs; ↑5-HTP	(Tian et al., 2020)
*B. breve* M-16V	CSDS male C57BL/6J mice 4 groups(n=11-22) each control, control with M-16V, CSDS, CSDS with M-16V	AIN-93G diet containing *B.breve* of 5.0×10^9^ nonviable cells/ 0.5 g daily	AIN-93G diet	33d	Suppress IL-1β increase in the PFC and HIP ↑Time in the interaction zone; ↓Time in the corner zone; ↓Bacteroidia; ↑*Bifidobacterium*	(Kosuge et al., 2021)
*A. muciniphila*	72 mice randomized to 8 groups donor CB (n= 10), donor CRS (n= 20), RE-CB (n = 6), RE-CRS (n = 6), RE-CRS-AKK (n= 6), RE-CB-DSS (n = 8), RE-CRS-DSS (n = 8), RE-CRS-AKK-DSS (n = 8)	100 μL of *A. muciniphila* containing 1×10^8^ bacteria daily	PBS	14d	Restore colonic mucus; Modify the gut microbiota; ↑α-diversity; *↑*Verrucomicrobia, *Akkermansia*, and *Ruminiclostridium*; ↓Immobility time in the TST and FST; ↑Movement distance in the OFT; ↑Colon length; ↓Histopathological scores	(Chen T. et al., 2021)
*A. muciniphila*	24 C57BL/6J mice 3 groups (n=8) each low-fat diet group, HFD group, HFD+P9 group 28 C57BL/6J mice WT: HFD (n=8), HFD+P9 (n=8), IL-6-KO: HFD (n=6), HFD+P9 (n=6)	P9 from *A.muciniphila* (100μg per mouse) daily	PBS	8w	↑GLP-1; ↑Uncoupling protein 1	(Yoon et al., 2021)
*A. muciniphila*	Exp1: female C57BL/6 mice (each group n=3-5) PBS group, BAA-835 group Exp2: female Lgr5-EGFP mouse (each group n=3-5) PBS group, PBS+R+M group, BAA-835+R+M group	*A.muciniphila* of 8 × 10^8^ CFU per dose daily	PBS	4w	Accelerate the proliferation of Lgr5+ ISCs; Promote the differentiation of paneth cells and goblet cells; ↑Acetic and propionic acids; Reduce gut damage; ↑Intestinal epithelial regeneration	(Kim S. et al., 2021)
*A. muciniphila*	36 C57BL/6 male mice, 6 groups (n=6) each CRS group, CRS+AKK group, AKK group, CRS+*Lactobacillus* L group, CRS+*Lactobacillus* H group, Control group	AKK (1×10^8^ CFU) *via* gavage	PBS	3w	↓GC; ↑BDNF; ↑DA; ↑OFT; ↓FST, TST; ↑The total distance; ↑β-alanyl-3-methyl-l-histidine; ↑Edaravone; ↑Verrucomicrobia; ↓Epsilonbacteraeota, Patescibacteria, Chlorofexi, and Acidobacteria	(Ding et al., 2021)
*C. butyricum* RH2	28 male adult SD rats 4 groups (n=7) each sham, stress, stress + RH2, positive control (Reserpine)	RH2 of 1 × 10^9^ CFU/ml/day/rat) by gastric gavage	vehicle	17d	↓ACTH; ↓CORT; ↑BDNF; ↓PAI-1; ↓FST; ↑The central area distance, the total distance, and the average speed	(Zhang W. et al., 2022)
*C. butyricum* WZMC1018	30 male C57BL/6J mice, 3 groups (n = 10) each control group, CUMS group, Cb group	Cb of 2.5×10^8^ CFU/day/mice	the equivalent milk	4w	↑5-HT; ↑BDNF; ↑GLP-1; ↑GLP-1R	(Sun et al., 2018)
*C. butyricum* miyairi 588	66 male C57BL/6 mice CBM588/stress group (n=25), placebo/stress group (n =25) the mice not exposed to CSDS (n=7 in the probiotics group, n=9 in the placebo group)	sterile water containing miyairi 588 (>5×10^6^/CFU)	sterile water	28d	↓IL-1β, IL-6, and TNF-α; *↑*Firmicutes; Relieve intestinal dysfunction and hippocampal microglial activation	(Tian T. et al., 2019)
EF2001	152 male ddY mice Sham group, OBX group, OBX+EF-2001 group	EF-2001 (250 mg/kg) once a day in a volume of 0.1 mL/10g mouse body weight	water	28d	↑Myelin and paranodal proteins; ↑p-cAMP-CREB expression; ↑p-NFκB p65 expression in astrocytes, p-signal transducer and activator of STAT3 expression; ↓Immobile time; ↑Grooming time; ↑BDNF; ↑LIF	(Takahashi et al., 2022)
EF2001	239 male ddY mice Exp1: Water group, DSS 1.5% group, DSS 1.5%+EF-2001 group, DSS 1.5%+DEX group Exp2: vehicle-treated water group, vehicle-treated DSS group	EF-2001 (250 mg/kg)	solvent	20d	↓TNF-α, IL-6; ↓Caspase-3; ↑Hippocampal neurogenesis ↑NF-κB p65 expression ↑XIAP	(Takahashi et al., 2019)
EC-12	Male C57BL/6J mice EC-12 group (n = 8), Control group (n = 8)	AIN-93M diet with heat-killed EC-12 at a concentration of 0.125%	AIN-93M basal diet	4w	↑OFT; ↑EPM; ↓FST; ↑Adrb3 and Avpr1a; ↑*Butyricicoccus* and *Enterococcus*	(Kambe et al., 2020)

5-HT, 5-hydroxytryptamine; 5-HTP, 5-hydroxytryptophan; A. muciniphila, Akkermansia muciniphila; ACTH, adrenocorticotropic hormone; B. dentium, Bifidobacterium dentium; C. butyricum, Clostridium butyricum; CBM588, Clostridium butyricum miyairi 588; CFSS, chronic foot shock stress; CLSTN1, Calsyntenin-1; CORT, corticosterone; CPE, Carboxypeptidase E; CRS, chronic restraint stress; CSDS, chronic social defeat stress; CUMS, chronic unpredictable mild stress; d, day; DA, dopamine; DAT, dopamine transporter; EC-12, Enterococcus faecalis EC-12; EF 2001, Enterococcus faecalis 2001; FOS/GOS, fructo-oligosaccharide and galactooligosaccharide; GABA, γ-aminobutyric acid; GC, Glucocorticoid; GLP-1, glucagon-like peptide-1; GR, glucocorticoid receptor; HFD, high-fat-diet; HPA, hypothalamic-pituitary-adrenal; IL-10, interleukin 10; IL-4, interleukin 4; IL-5, interleukin 5; ISCs, intestinal stem cells; JAK/STAT, The Janus kinase/signal transducers and activators of transcription; Klf4, Krüppel-like factor 4; LIF, leukemia inhibitory factor; LPS, lipopolysaccharide; m, month; MAPK, mitogen-activated protein kinase; miR, microRNAs; NA, noradrenaline; NE, noradrenaline; NMDAR, N-methyl-D-aspartic acid receptor 1; OFT, open field test; PAI-1, plasminogen activator inhibitor 1; pCREB, phosphorylated cAMP-response-element-binding; SCFA, short-chain fatty acid; Tff3, Trefoil factor 3; tNAA, total N-acetylaspartate; TNF-α, tumor necrosis factor-α; TrkB, tyrosine kinase receptor B; TST, tail suspension test; w, week; XIAP, X-linked inhibitor of apoptosis protein; ZO-1, zonula occludens 1.

/: no found; ↓: downregulation; ↑: upregulation.

### 4.1 Lactobacillus

*Lactobacillus* is one of the most widely used and intensively studied probiotic bacteria in gut microbiota. *Lactobacillus* spp. are anaerobic, gram-positive, peroxidase-negative, non-spore-forming rods that grow better under the microaerobic condition. As one of the inhabitants of the healthy microbiota in the human gut, vagina, and oral cavity, *Lactobacilli* have been considered safe microorganisms for the host health, with low pathogenic potential, and lack the ability to transmit antibiotic resistance factors to pathogens (Saarela et al., 2000). Thus, *Lactobacilli* strains isolated from natural products have been proposed as promising probiotic candidates. Several *Lactobacilli* strains have been used as probiotics, including *L. plantarum*, *L. fermentum*, *L. rhamnosus* and *L. casei*, which are isolated from the gut and exert various benefits to the host, including attenuation of anxiety and cognitive improvement (Goldstein et al., 2015). Many previous studies have demonstrated the beneficial effects of these *Lactobacilli* strains on mood, anxiety, and cognition, which can be considered as potential psychobiotics.

Among these *Lactobacilli* strains, *L. rhamnosus* must be mentioned, which is acid- and bile-stable and has a strong affinity for human intestinal mucosal cells. Many animal studies have found that oral administration with one *L. rhamnosus* strain, *L. rhamnosus* JB-1 (JB-1), demonstrates psychoactive and neuroactive properties. JB-1 can change the levels of neurotransmitters in the brains of mice, which in turn reduces stress-induced anxiety- and depression-related behaviors (Bravo et al., 2011; Janik et al., 2016). JB-1 consistently regulates the expression of GABA_A_ and GABA_B_ receptors in a region-dependent manner in mice, restoring metabolites such as GABA and glutamate to normal levels and reducing corticosterone levels (Tette et al., 2022). Janik et al. reported that 25% of central GABA levels could be elevated by four weeks of treatment with the JB-1 strain in BALB/c mice (Janik et al., 2016). Interestingly, the antidepressant effects of JB-1 depend on the intact vagus nerve connection between the gut and brain (Bravo et al., 2011). Subphrenic vagotomy prevented this effect, suggesting that the modulatory effect of JB-1 on GABA proceeds through the vagus nerve. JB-1 can directly stimulate vagal afferent neurons in the gut, with signals uploaded to the solitary bundle nucleus, followed by projections to the paraventricular nucleus, ultimately activating the GABAergic system, creating negative feedback, and lowering cortisol levels (Bravo et al., 2011). In addition, JB-1 also results in modulating the immune system and induces regulatory T cells, which have been found to be both necessary and sufficient to mediate the behavioral effects of bacteria (Liu et al., 2020). However, JB-1 can attenuate stress-induced behavioral deficits successfully but fails to re-establish the diversity and richness of the gut microbiota or correct the relative abundances of specific bacteria that altered by stress. This suggests that the neuroactive properties of beneficial microbes may not be mediated by gut microbiota restoration, but be determined by their functional activity (bacterial metabolites) and direct modulation of host signaling pathways (Bharwani et al., 2017).

Another *L. rhamnosus* strain, *L. rhamnosus* GG (LGG), which originates indigenously in the human gut, became available for use as a probiotic in Finland in 1990. LGG colonization early in life increases tight junction protein expression and immunoglobulin A production, upregulates host immune responses, increases intestinal villus length and colonic crypt depth, and enriches beneficial bacteria such as *Bifidobacterium* and *Akkermansia* (Zhou et al., 2022). With its effects on the increase in SCFAs-producing bacteria such as *Bifidobacterium*, mice had significantly higher levels of acetate, which helped alleviate anxiety (Strati et al., 2016). LGG has been found to increase GABA concentrations in fermented adzuki bean milk under optimized culture conditions (Song and Yu, 2018). In addition, LGG implantation can also activate epithelial growth factor receptor expression, enhance serotonin transporter protein expression, modulate the serotonergic system in the gut, and increase the levels of BDNF and GABA receptors in the amygdala and hippocampus (Cui et al., 2014; Johnson and Foster, 2018), which can alleviate anxiety and depression symptoms (Xu J. et al., 2022). Neufeld et al. observed that dietary supplementation with the probiotic LGG alone or in combination with the prebiotics polydextrose and galactooligosaccharide can ameliorate stress-induced increases in anxiety-like behavior (Mcvey Neufeld et al., 2019). However, the anti-depressive benefits are dependent on live LGG, while treatment with the heat-inactivated form of LGG had no effect.

Two other strains of *Lactobacillus*, *L. rhamnosus* CCFM1228 and *L. paracasei* CCFM1229, can alleviate anxiety- and depression-related behaviors in animal models, which may be achieved by regulating the activity of xanthine oxidase (XO) in brain (Xu M. et al., 2022). In fact, several anxiety- and depression-related indicators such as immobility time in the forced swimming test (FST), serum corticosterone level, and hippocampal BDNF concentration were significantly associated with XO activity in the cerebral cortex. XO activity is significantly increased in patients with depression, and xanthine and XO produce superoxide anions and free radicals that generate oxidative stress, leading to cellular damage and death. Xanthines synthesized *via* the pentose phosphate pathway can cross the BBB easily to the amygdala and act on oligodendrocytes *via* purine receptors on the cell surface, causing abnormal activation and proliferation of oligodendrocytes, leading to local neuronal hyperactivation in the fear center. *L. paracasei* CCFM1229 significantly upregulated the expression of Grin1, Grin2a, and Grin2b, and enhanced synaptic plasticity in depressed mice (Xu M. et al., 2022). It could also maintain the structural and functional stability of myelin by upregulating Mbp mRNA expression. Myelin loss and oligodendrocyte dysfunction may be involved in depression pathogenesis. In contrast to LGG, both live and heat-killed *L. paracasei* PS23 can reverse chronic corticosterone-induced anxiety- and depression-like behaviors (Wei et al., 2019). *L. rhamnosus* CCFM1228 can enhance astrocyte function in depressed mice by upregulating Gfap mRNA expression significantly. *L. rhamnosus* CCFM1228 significantly downregulates CD36 mRNA expression, which is upregulated in depressed mice, and CD36 deficiency may influence depression-like behavior by altering the gut microbiota and inflammatory pathways (Xu M. et al., 2022). In addition, taurine deficiency may lead to oxidative stress as well as reduced total N-acetylaspartate levels in neurodegenerative diseases, which can be restored by supplementation with *L. rhamnosus* JB-1 (Devkota et al., 2012; Kochalska et al., 2020). Of course, other *Lactobacillus* strains, alone or in combination with other microorganisms, have also been investigated for their roles and possible mechanisms in anti-depressive behavior.

### 4.2 Bifidobacterium

Like *Lactobacillus*, *Bifidobacterium* is a commonly used probiotic bacterium. *Bifidobacterium* spp. is a genus of gram-positive, non-motile, rod-shaped, sometimes bifurcated at one end, strictly anaerobic bacteria that are widely found in the human and animal digestive tract. Most probiotic *Bifidobacterium* strains have shown positive effects on human health. *Bifidobacterium* inhibits the proliferation of harmful bacteria, ameliorates the function of the gastrointestinal mucosal barrier, and protects against pathogens. With the growing recognition of the existence of the MGB axis, recent studies have identified that *Bifidobacterium* can affect the functioning of the brain and CNS, leading to alterations in behavior, and cognitive abilities of humans and animals. Aizawa et al. found that lower fecal counts of *Bifidobacterium* in depressed patients than that in healthy controls, suggesting that *Bifidobacterium* plays an important role in the pathogenesis of depression (Aizawa et al., 2016). In GF rats, *Bifidobacterium* can successfully inhibit elevated HPA axis and depression-like behaviors (Messaoudi et al., 2011). Kazem et al. demonstrated that a significant improvement in depression and well-being status was obtained after the administration of probiotic *Bifidobacterium* spp. for 8 weeks (Kazem et al., 2021).

Several strains of *Bifidobacterium* such as *B. adolescentis*, *B. dentium*, and *B. infantis* exert beneficial effects in reducing anxiety- and depression-like behaviors, which are related with the production of GABA. These GABA producers can bioconvert monosodium glutamate to GABA (Barrett et al., 2012). *Bifidobacterium* can biosynthesize GABA from glutamate by the action of glutamate decarboxylase, and then transport it extracellularly by the action of the glutamate GABA antiporter. One efficient GABA producer, *B. adolescentis* 150, can attenuate depression-like behavior during the FST conducted on BALB/c mice (Yunes et al., 2020). Guo et al. also found that *B. adolescentis* exhibits antidepressant and anxiolytic effects, which are associated with a reduction in inflammatory cytokines and re-establishment the gut microbiota (Guo et al., 2019). Another *Bifidobacterium* strain, *B. breve* CCFM1025, exerts an antidepressant-like effect by reshaping the gut microbiota, increasing the production of beneficial metabolites, attenuating the HPA axis and inflammation, upregulating BDNF expression, and downregulating c-Fos expression in the brain (Tian et al., 2020). In one RCT, Tian et al. observed that *B. breve* CCFM1025 can attenuate depression and associated gastrointestinal disorders by altering the gut microbiota and gut tryptophan metabolism (Tian et al., 2022), suggesting that *B. breve* CCFM1025 is a promising psychobiotic candidate. Interestingly, *B. breve* CCFM1025 can normalize the abundance of SCFA-producing species such as *Heterobacterium* spp., *Clostridium faecium*, and *Clostridium tumefaciens* in patients with depression, whereas *B. longum* subsp. *infantis* E41 significantly reduced the elevated abundance of Veillonellaceae and *Desulfovibrio* (Tian et al., 2022). 5-hydroxytryptophan (5-HTP), a precursor substance of 5-HT and a key neurotransmitter that can cross the BBB, links bidirectional gut-brain communication, making it possible for the brain and gut to maintain a host’s health jointly. Intestinal and serum 5-HTP were positively correlated with brain 5-HT levels. Tian also found that *B. breve* M2CF22M7 and *B. longum* subsp. *infantis* E41 have antidepressant effects in mice, partly by improving the secretion of 5-HTP, reversing the deficits in hippocampal 5-HT, and increasing BDNF levels in the prefrontal cortex (Tian P. et al., 2019). A previous study also found that chronic stress impaired the negative feedback of corticosterone in the HPA axis by down-regulating the glucocorticoid receptors (Nr3c1), leading to glucocorticoid resistance, which is also coincident with a high level of inflammation. *B. breve* CCFM1025 normalizes the expression of Nr3c1 to reduce cortisol levels (Tian et al., 2020). In addition, the presence of glutamate decarboxylase genes in *Bifidobacteria* may mediate GABA production, which can complement GABA levels and enhance the negative feedback regulatory mechanism of cortisol, as exemplified by *B. adolescentum* (Duranti et al., 2020). Higher levels of pro-inflammatory cytokines lead to the onset and progression of depression-related behaviors, whereas *Bifidobacteria* are known for their ability to counter inflammation (Dyakov et al., 2020). An increase in pro-inflammatory cytokines, namely IL-6, IL-1β, tumor necrosis factor α, and C-reactive protein (CRP), participate actively in the development of depression. *B. bifidum* TMC3115 suppresses the stress-induced increase in IL-6 levels and reduces inflammation, which can alleviate stress-induced inflammatory responses (Yoda et al., 2022). In addition, *Bifidobacterium* can promote the production of Foxp3^+^ Tregs mediated by SCFAs to limit the inflammatory response in peripheral tissues (Arpaia et al., 2013; Smith et al., 2013). In contrast to the findings in LGG, Kosuge et al. observed that heat-sterilized *B. breve* M-16V can prevent depression-like behavior and IL-1β expression induced by chronic social defeat stress through modulation of gut microbiota composition in mice (Kosuge et al., 2021). These findings suggest that *Bifidobacterium* could be used as a potential psychobiotics for the prevention and treatment of depression.

### 4.3 Akkermansia muciniphila

*A. muciniphila* is a gram-negative anaerobic bacterium, representative of the phylum Verrucomicrobia, a symbiotic bacterium widely distributed in the mucus layer of the human intestine, which lives by breaking down mucin (Derrien et al., 2008). Due to its beneficial effects in many diseases, it has attracted much attention and research from the academic community and has become a new generation of probiotics. Several studies have shown that *A. muciniphila* can influence glucose and lipid metabolism, and intestinal immunity, while certain food ingredients, like polyphenols, can increase the abundance of *A. muciniphila* in the gut. The abundance of *A. muciniphila* is significantly reduced under certain medical conditions. *A. muciniphila* is negatively associated with DM and obesity. Recent studies indicate that several neurological disorders, including amyotrophic lateral sclerosis, depression, AD, and ASD, disrupt the abundance of *A. muciniphila* (Wang et al., 2011; Blacher et al., 2019; Li B. et al., 2019; Mcgaughey et al., 2019). *A. muciniphila* has emerged as the “sentinel of the gut”, which can promote gut barrier integrity, enrich butyrate-producing bacteria modulate immune responses, and inhibit inflammation (Ouyang et al., 2020). Given its higher abundance in healthy mucosa, *Akkermansia* has been suggested as biomarker of healthy intestines (Mcgaughey et al., 2019).

*A. muciniphila* feeds on mucin in the mucus layer of the intestine, thereby settling in the intestine and protecting it from pathogens *via* competitive rejection (Remely et al., 2015; Kim et al., 2022). Colonic mucus is heavily dependent on the release of MUC2, a hydrated glycosylated protein produced by cupped cells that adheres to the colonic surface to prevent invasion by luminal microbes and pathogens (Mcgaughey et al., 2019). *A. muciniphila* can improve MUC2 expression and increase the number of cupped and MUC2 cells to strengthen the colonic mucosal barrier in recipient mice (Chen T. et al., 2021). At the same time, *A. muciniphila* breaks down mucin to produce SCFAs, such as acetate and propionate, to play a regulatory role. SCFAs can alleviate oxidative stress and inflammatory responses through the GLP-1 pathway, as well as act in intestinal Treg homeostasis or directly through the BBB to increase the levels of neurotransmitters such as glutamine, glutamate, and GABA in the hypothalamus of mice, exerting an antidepressant effect (Zhai et al., 2019). In socially defeated animals, the decrease of *Akkermansia* spp. was correlated with the behavioral metrics of both anxiety and depression positively. McGaughey et al. found that reduction of fecal *Akkermansia* spp. in mice exhibited decreased center time during the open field test (OFT), indicating increased anxiety-like behavior, as well as decreased sucrose preference, suggesting increased anhedonia (Mcgaughey et al., 2019). Generally, supplementation with *A. muciniphila* may improve gut dysbiosis due to depression. Ding et al. found that *A. muciniphila* reduces depression-like behaviors induced by chronic stress by regulating gut dysbiosis and metabolic disorders related to the gut microbiota (Ding et al., 2021). *A. muciniphila* increases the level of *Akkermansia* and decreases the relative abundance of *Helicobacter*, *Lachnoclostridium*, *Candidatus_Saccharimonas*, and *Eubacterium_brachy*, promoting the re-establishment of gut microbiota (Ding et al., 2021). Increased *Clostridium tumefaciens* abundance after *A. muciniphila* treatment is negatively correlated with the number of microglia, which may exert an anti-neuroinflammatory effect. In addition to gut microbiota modulation, the antidepressant effect of *A. muciniphila* positively correlates with an increase in metabolites, such as edaravone and β-alanyl-3-methyl-l-histidine (Ding et al., 2021). Edaravone increased serotonin concentrations significantly, whereas β-alanyl-3-methyl-l-histidine increased the levels of dopamine (Ding et al., 2021). *A. muciniphila* administration may also reduce the degradation of geraniol, which has neuroprotective and anti-inflammatory activities and may alleviate depression (Deng et al., 2015; Ding et al., 2021). In addition, Amuc_1100, the outer membrane protein of *A. muciniphila*, also plays an important and direct role in the crosstalk between *A. muciniphila* and its host. Cheng et al. found that Amuc_1100 can improve chronic unpredictable mild stress (CUMS)-induced depression-like behavior and CUMS-induced downregulation of serotonin in the serum and colon of mice, restore gut dysbiosis, upregulate BDNF, and inhibit inflammation in the hippocampus (Cheng et al., 2021). Recently, they also found that the Amuc_1100^Δ80^, a truncated protein with 80 amino acids truncated at the N-terminus of Amuc_1100, shows a better antidepressant effect on modifying CUMS-induced depression-like behavior in mice than Amuc_1100 does (Cheng R. et al., 2022). Taken together, both human and animal studies have consistently reported that increasing *A. muciniphila* abundance can be a potential method for treating depression- and anxiety-like behaviors. Direct and compelling evidence from future comprehensive pre-clinical analyses and well-designed clinical studies is necessary to explore the therapeutic potential of *A. muciniphila* in depression.

### 4.4 Clostridium butyricum

*Clostridium butyricum* is a gram-positive anaerobic bacterium that can generate SCFAs by consuming undigested dietary fiber, mainly butyrate and acetate. It has a strong ability to survive independently of stomach and bile acids. *C. butyricum* plays an important role in regulating the gut microbiota, which has been safely used as a probiotic for decades. It is increasingly utilized in the treatment of various human diseases, including intestinal injury, gut-acquired infection, IBS, IBD, neurodegenerative diseases, metabolic diseases, and colorectal cancer (Lynch and Pedersen, 2016). Generally, *C. butyricum* promotes the proliferation of beneficial *Bifidobacteria*, *Lactobacilli*, and anthropoid bacteria, reducing pathogens and providing benefits to the intestinal microbial ecosystem (Hagihara et al., 2018; Miao et al., 2018).

Previous studies have found that exposure to chronic or acute psychosocial stress decreases the level of *Clostridium* spp. in the cecum (Bailey et al., 2011), while mental stress increases the levels of peripheral IL-6 and chemokine CCL2, and the expression of TLR3 and TLR4 in the prefrontal cortex of suicidal patients with depression, leading to hyperactivation of microglia (García Bueno et al., 2016; Park et al., 2018). Recently, many preclinical studies have shown that *Clostridium* spp. alone or in combination with other antidepressants can be used to treat depression (Sun et al., 2018; Tian T. et al., 2019). Liu et al. found that *C. butyricum* can be considered as a safe and economical therapeutic option to treat mental disorders, which can influence the gut microbiota-butyrate-brain axis in mice (Liu et al., 2015). A specific phenotype of *C. butyricum*, *C. butyricum* MIYAIRI 588 (CBM588), was isolated from the feces of a Japanese person firstly. CBM588 has been used as a probiotic in humans and domestic animals, exerting a variety of beneficial health effects. Hagihara et al. first reported that the administration of CBM588 improved the ecosystem of the gastrointestinal tract in mice significantly, modulating the gut microbiota composition, increasing the numbers of *Bifidobacterium*, *Coprococcus*, and *Bacteroides*, enhancing butyrate production, and reducing epithelial damage (Hagihara et al., 2018). They also found that CBM588 treatment caused a functional shift of the gut microbiota toward increased carbohydrate metabolism (Hagihara et al., 2019). Tian et al. found that 28-day preventive treatment with CBM588 improved depression-like behaviors in mice with chronic social defeat stress (Tian T. et al., 2019). CBM588 can alter the composition of the gut microbiota, induce a higher abundance of *C. perfringens*, and produce more butyrate, exerting a regulatory effect of SCFAs on the MGB axis and reducing depression-like behavior. Simultaneously, CBM588 prevents stress-induced activation of hippocampal inflammatory microglia by reducing cytokines, including IL-1β, IL-6, and TNF-α (Tian T. et al., 2019). In a model of CUMS-induced depression, Sun et al. observed that the administration of *C. butyricum* CGMCC 9830 reversed depression-like behavior, increased hippocampal BDNF and 5-HT levels, and improved intestinal GLP-1 levels (Sun et al., 2018). GLP-1 is secreted by intestinal L cells distributed in the ileum and colon, and changes in the gut microbiota can affect GLP-1 levels, which are closely related to CNS function (Reimann et al., 2008). By producing metabolic butyrate, *Clostridium* spp. has been implicated in depression through HPA-axis perturbation and damage to intestinal permeability by combining G-protein receptor 43 (GPR43) and GPR41 and regulating NF-κB and PPARγ signaling (Lührs et al., 2002; Van De Wouw et al., 2018). In a recent prospective open-label trial, Miyaoka et al. found that subjects with treatment-resistant major depressive disorder (MDD) receiving CBM588 (60 mg/d) in combination with antidepressants (flvoxamine, paroxetine, escitalopram, duroxetine, and sertraline) reported significantly lower median scores across several indices (BDI, HAMD-17, and BAI scores) than those in the control group (Miyaoka et al., 2018). Although these preclinical and clinical studies have shown the efficacy and safety of *C. butyricum* in depression, future larger-scale RCT on depression should be conducted to provide clearer recommendations for *C. butyricum* application and evaluate its possible mechanisms.

### 4.5 Enterococcus faecalis

*Enterococcus faecalis* is a gram-positive, facultative anaerobic, lactic acid bacterium belonging to the phylum Firmicutes. *E. faecalis* is a normal resident of the gut in many hosts (García-Solache and Rice, 2019) and is generally believed to be harmless. Some strains of *E. faecalis* with beneficial effects are used as probiotics and starter cultures in the dairy industry, whereas other strains of *E. faecalis* contribute to the development of nosocomial infections and cause bacteremia, endocarditis, or urinary tract infections. Strain-specific differences in probiotic, pathogenic, and commensal *E. faecalis* may depend on their interaction with the host. Thus, *E. faecalis* has received substantial attention owing to its ‘dualistic’ behavior toward human health.

*E. faecalis* 2001 (EF-2001), one biogenic lactic acid bacterium, has been used as a probiotic to improve immunity and exert antitumor activity in mice (Choi et al., 2016; Gu et al., 2017). Takahashi et al. demonstrated that 20-day administration of EF-2001 can prevent colitis-induced depression-like behavior *via* the MGB axis in mice, which can reduce rectal and hippocampal inflammatory cytokines such as TNF-α and IL-6 and facilitate the NF-κB p65/XIAP pathway in the hippocampus (Takahashi et al., 2019). Olfactory bulbectomized (OBX) mice is a valuable experimental animal model for MDD, which expresses various depression-like behaviors such as anhedonia, memory impairment, and reduction in sexual contact (Takahashi et al., 2018). Recently, EF-2001 was shown to prevent OBX-induced depression-like behaviors through the regulation of prefrontal cortical myelination *via* the enhancement of CREB/BDNF and NF-κB p65/LIF/STAT3 pathways (Takahashi et al., 2022). In addition, this group also found that the anti-dementia effects of EF-2001 in OBX mice are associated with the enhancement of hippocampal neurogenesis *via* the ERK-CREB-BDNF pathway (Takahashi et al., 2020). Another Japanese group observed that male mice fed a diet supplemented with heat-killed *E. faecalis* strain EC-12 showed decreased anxiety-like behavior in OFT and elevated plus-maze test, which can increase the expression of neurotransmitter receptor genes such as Adrb3, Avpr1a, and Drd5, and improve the gut dysbiosis (Kambe et al., 2020). These findings suggest that some strains of *E. faecalis* have the potential to alleviate depressive symptoms. Future studies are required to explore the antidepressant effects and the exact mechanism of action of *E. faecalis* in the human brain.

## 5 Probiotic treatment for depression-related comorbidities

A large body of evidence has demonstrated that depression is not only more common with other psychiatric disorders; for example, up to 90% of patients with anxiety disorders present with co-morbid depression (Tiller, 2013), but it is also highly comorbid and occurs together with many physical diseases, such as IBS, IBD, and metabolic syndromes such as DM and obesity. A common pathological mechanism of co-morbidity is a prerequisite for the administration of the same drug to achieve common remission. To understand if probiotics have this potential, we have addressed recent advances in the application of probiotics in depression-related disorders, which will provide novel therapeutic options for these co-morbid disorders associated with depression.

### 5.1 Irritable bowel syndrome

IBS is a chronic dysfunction of the gastrointestinal system characterized by altered bowel habits and abdominal pain in the absence of biochemical or structural abnormalities, primarily manifesting as diarrhea, constipation, or both. IBS affects approximately 7% to 21% of the global population (Chey et al., 2015). Based on the Rome IV criteria, which was updated in June 2016, the gold standard for the diagnosis of IBS is the exclusion of other diseases (Mearin et al., 2016). Patients with IBS are categorized into four subtypes based on their predominant stool habits: IBS-C (constipation, 20-30%), IBS-D (diarrhea, 38-50%), IBS-M (mixed type, 6-16%), and IBS-U (unclassified, 24-60%). The pathophysiology of IBS is not fully understood, but evidence suggested that abnormalities in the composition or metabolic activity of the gut microbiota are associated with its progression (Han et al., 2022). Increasing studies have suggested that the MGB axis plays a role in IBS.

IBS is frequently associated with psychiatric comorbidities such as depression and gut-brain crosstalk is thought to contribute to its development (Ray, 2017). A significant association between IBS and MDD has been reported previously, and most patients with IBS identify stress and anxiety as symptom aggravators (Lacy et al., 2007). Lee et al. reported significantly higher levels of depression and anxiety in patients with IBS than in healthy controls (Lee et al., 2017). Midenfjord et al. also found that patients with IBS who suffered from psychological distress reported more severe gastrointestinal symptoms (Midenfjord et al., 2019). Recent evidence suggests that gut dysbiosis can be considered one of the fundamental theories that can explain both physical and mental symptoms in patients with IBS. Alterations in the gut microbiota, MGB axis, and neuro-immune system may be the cornerstone of the association between IBS and depression (Mudyanadzo et al., 2018). A systematic review identified that patients with comorbid IBS and anxiety/depression had lower α-diversity, higher levels of Proteobacteria, Prevotella/Prevotellaceae, and Bacteroides, and lower abundance of Lachnospiraceae than the controls (Simpson et al., 2020). This suggests that microbiota modulation with specific probiotics or other microecological regulators may be a useful therapeutic approach for depression-related disorders, such as IBS.

In fact, recent findings suggest that probiotics may improve host health in patients with IBS both physically and mentally (Le Morvan De Sequeira et al., 2021; Zhang T. et al., 2022). The safety and efficacy of probiotics in the treatment of IBS are supported by numerous clinical studies. A systematic review and network meta-analysis of 43 RCTs involving 5,531 IBS patients provided data regarding the best probiotic species used in the treatment of IBS (Zhang T. et al., 2022). Several probiotic strains, such as *B. bifidum*, *B. infantis*, *L. casei*, *L. acidophilus*, *L. plantarum*, *Bacillus coagulans*, *C. butyricum*, and *Saccharomyces boulardii* alone or in combination, have been used to treat depression in patients with IBS. Among these probiotic strains, *B. coagulans* exhibited the highest probability of being the optimal probiotic species for improving IBS symptom relief rate, as well as global symptoms, bloating, abdominal pain, and straining scores. *L. plantarum* ranked first in improving the QoL of patients with IBS and had the lowest incidence of adverse events. *B. coagulans* can promote intestinal digestion, maintain host microbiota homeostasis, and regulate the host immune system, and has been studied in the treatment of several human diseases. Recently, a double-blind RCT reported that *B. coagulans* MTCC 5856 as a single probiotic agent at a dose of 2 × 10^9^ spores (CFU) per day showed robust efficacy in the treatment of patients with IBS symptoms and MDD (Majeed et al., 2018). *B. coagulans* MTCC 5856 could improve the Montgomery-Asberg Depression Rating Scale (MADRS), and Hamilton Rating Scale for Depression (HAM-D) scores, indicating that it may be a new optional approach for the management of depression in patients with IBS. *B. coagulans* MTCC 5856 can produce SCFAs (such as acetic, propionic, and butyric acid), neurotransmitters, and antimicrobial and anti-inflammatory substances, which could be the possible mechanism of action in alleviating depression symptoms. In addition, this probiotic significantly reduced the level of myeloperoxidase (an inflammatory biomarker), which is responsible for the production of free radicals. Another placebo-controlled trial observed that the probiotic *B. longum* NCC3001 reduced depression, but not anxiety scores, and increased the QoL in patients with IBS (Pinto-Sanchez et al., 2017). These improvements were associated with changes in the brain activation patterns, indicating that this probiotic reduces limbic reactivity. Despite these promising findings, there is still limited evidence for the efficacy of probiotic intervention in patients with IBS and depression, as the benefit of probiotics tends to be symptom- and strain-specific. Further prospective, larger-scale trials with extended follow-up durations, as well as a detailed assessment of the therapeutic effects of specific probiotic supplementation, are critical prior to managing depression in patients with IBS with probiotics in clinical practice.

### 5.2 Inflammatory bowel disease

IBD, including Crohn’s disease (CD) and ulcerative colitis (UC), is an idiopathic, lifelong, and destructive chronic inflammatory condition of the gastrointestinal tract that affects tens of millions of people worldwide (Mulder et al., 2014). The pathogenesis of IBD is incompletely understood, although the major factors influencing IBD may include genetic, environmental, and microbial determinants (Jakubczyk et al., 2020). Recently, gut microbiota has been increasingly recognized as a critical and central factor in IBD. There is a growing consensus that inappropriate activation of the immune system by commensal bacteria underlies IBD.

The disease burden of IBD includes not only the physiological manifestations of the disease but also psychological and social burdens. Although the link between IBD and psychological disorders remains unclear, patients with IBD have a high prevalence of depression and anxiety. Psychological symptoms appear to be more prevalent in active disease states, with no difference in prevalence between CD and UC. The comorbid prevalence of depressive symptoms was 25.2%, and 38.9% of patients with active IBD had depression (Barberio et al., 2021). Compared to those who do not show psychiatric symptoms, patients with IBD suffering from depression have a decreased remission, and patients demonstrating depression show a more severe illness over a longer period of time (Stasi and Orlandelli, 2008). Chen et al. have summarized that depression in IBD may arise through an “IBD-inflammation-kynurenine pathway-depression” association (Chen LM. et al., 2021). Thus, encouraging screening and treatment of these comorbid psychiatric disorders may improve the prognosis of patients with IBD.

Gut dysbiosis is commonly observed in IBD patients with depression, generally with increased Firmicutes and reduced Proteobacteria (Chen DL. et al., 2021; Yuan et al., 2021). Weis et al. also observed that disorganized gut microbiota and disturbed metabolism were found in patients with active UC accompanied by depression and anxiety, which could increase systematic inflammation (Weis et al., 2019). The altered gut microbiota in patients with IBD can disturb bidirectional communication in the gut-brain axis, which might be associated with potential consequences for the CNS. The gut dysbiosis in IBD patients with depression represents a potential therapeutic target. The modulation of the gut microbiota using probiotics can alter the behavioral response in IBD, which has been increasingly studied in mouse models. Mice subjected to dextran sodium sulfate (DSS)-induced acute IBD-like colitis demonstrate behavioral changes, including anxiety-like behaviors and cognitive deficits. In DSS-induced animal models, probiotics, such as *B. longum* NCC3001, can reduce anxiety-like behaviors induced in rats in response to DSS-induced colitis (Bercik et al., 2011b). The probiotic *B. longum* NCC3001 normalizes behavior by decreasing the excitability of enteric neurons, but does not affect MPO activity, histological scores, or BDNF levels. In another previously mentioned behavioral study, Emge et al. found that recognition memory deficits and anxiety-like behavior during acute inflammation in murine IBD were improved by the administration of a probiotic mixture containing *L. rhamnosus* R0011 and *L. helveticus* R0052 (Emge et al., 2016). The improvements in behavior after probiotic administration were broadly correlated with the restoration of the microbiota and modulated hippocampal c-Fos expression. Inflammation may serve as a common trigger for the altered cognitive function observed in these models. Yoo et al. found that oral administration of a probiotic mixture containing *L. plantarum* NK151, *B. longum* NK173, and *B. bifidum* NK175 could also alleviate stress-induced fatigue, depression, and IBD by modulating inflammatory cytokines and gut microbiota byproducts such as LPS (Yoo et al., 2022). A previous study found that *E. coli* K1 significantly caused psychiatric disorders, such as depression and memory impairment, and IBD-like colitis in SPF and GF mice (Jang et al., 2018). Yun et al. observed that oral gavage of *L. gasseri* NK109 significantly alleviated *E. coli* K1-induced depression-like behaviors in GF and SPF mice by regulating the immune response through NF-κB-mediated BDNF expression, IL-1β expression, and vagus nerve-mediated gut-brain signaling (Yun et al., 2020). In addition, this group demonstrated that *L. reuteri* NK33 and *B. adolescentis* NK98 synergistically improved the occurrence and development of anxiety/depression and colitis by regulating gut immune responses and microbiota composition (Jang et al., 2019). However, *E. faecium* and *Pediococcus acidilactici*, known as probiotic strains, deteriorated Enterobacteriaceae-induced depression and colitis in mice (Jang et al., 2022). Another probiotic strain, *Weissella paramesenteroides* WpK4, can reduce anxiety-like and depression-like behaviors in murine models of ulcerative colitis by regulating the MGB axis and reducing gut permeability (Sandes et al., 2020). One Chinese group also observed that *L. plantarum* DMDL 9010 intake could reduce colitis and depression-like behavior in mice with DSS-induced colitis by upregulating the levels of neurotransmitters (especially 5-HT, DA, NE, and 5-HIAA) and SCFAs (such as butyric acid and propionic acid) (Huang et al., 2022). Considering these encouraging data from preclinical trials, microbiota modulation with probiotics might offer a novel therapeutic approach to combat behavioral comorbidities, such as depression, in patients suffering from IBD. However, the benefits of these treatments are limited because of the scarcity of interventional studies. Further well-designed clinical trials should be conducted to confirm the benefits of these probiotics in patients with IBD and comorbid depression.

### 5.3 Metabolic syndrome

Metabolic syndrome is defined as a cluster of obesity, hypertension, dyslipidemia, and dysglycemia. Over the past few decades, the prevalence of metabolic syndrome has increased markedly worldwide, which may be explained by urbanization, aging, lifestyle changes, and nutritional transition. Metabolic syndrome has become a serious public health problem, highlighting the urgent need to tackle metabolic syndrome in China and other populations (Li et al., 2018). Obesity is defined as abnormal or excessive fat accumulation that presents a risk to health and is a well-known cause of cardiovascular disease burden and premature death. DM is a chronic disease caused by an inherited or acquired lack of insulin produced by the pancreas or the inability of the body to fully utilize the insulin produced. A growing body of evidence suggests that co-morbid metabolic syndrome and depression are common and are often considered “metabolic depression” (Demakakos et al., 2010; Forsythe et al., 2010). Major depression and the exacerbation of depression symptoms have been reported in 11% and 31% of patients with DM, respectively (Semenkovich et al., 2015). The lifetime prevalence of major depression is approximately 28.5%. It has been reported that the prevalence of depression in patients with diabetes is higher than in the normal population (Anderson et al., 2001). In a meta-analysis of 17 community-based studies with 204,507 participants, there was a significant association between depression and obesity (De Wit et al., 2010). There is evidence of a dose-response effect of obesity severity on the odds of depression (Onyike et al., 2003). Obesity increases the incidence of depression as a function of metabolic dysfunction (Opel et al., 2015; Delgado et al., 2018). Depression is linked to a higher rate of complications in metabolic syndrome, to more disability, and to loss of years of life. Depression worsens glycemic control and causes greater severity of DM complications, resulting in poorer adherence to DM self-care (adherence to diet, checking blood sugar level), increased risk of retinopathy and macrovascular complications, decreased QoL, and increased disability burden (De Groot et al., 2016). Obesity leads to poorer performance in diverse cognitive tasks, and these deficits are exacerbated in instances of comorbid depressive disorder (Restivo et al., 2017). Thus, a better understanding of the link between comorbid depression and metabolic syndrome is critical to inform appropriate preventive and intervention strategies. Simultaneous therapeutic option for depression and metabolic syndrome is merited to enhance treatment outcomes in both conditions.

The exact mechanisms underlying the association between depression and metabolic syndrome are poorly understood, and possible pathophysiological overlap has been proposed. However, whether there is a causal relationship between both diseases and the nature of that causal relationship is still unclear. Recent advances have reported that both depression and these metabolic diseases are related to chronic low-grade inflammation and gut dysbiosis (Chan et al., 2019). The gut microbiota plays a vital role in regulating both the metabolic and brain functions of the host by reducing inflammatory activation and affecting the regulation of energy balance and release of neurotransmitters, suggesting that the gut microbiota can be considered a promising target to treat metabolic depression. To date, there have been no specific pharmacotherapies for metabolic depression. Current therapies for both depression and metabolic syndrome remain suboptimal for many patients, thus making improvements and advances in intervention options in high demand. Increasing evidence indicates that probiotics play a promising role in the management of these comorbidities. Probiotics appear to be effective in reducing depressive symptoms and can improve some of the clinical components of metabolic syndrome, making it a potential new therapeutic option or patient-specific strategy to treat both metabolic and depressive disorders.

Numerous animal and clinical studies have provided evidence for the treatment of metabolic depression with different probiotics. Patterson et al. found that daily administration of GABA-producing *L. brevis* DSM32386 and *L. brevis* DPC6108 ameliorated both metabolic abnormalities and depression-like behavior associated with metabolic syndrome in mice (Patterson et al., 2019). Specifically, *L. brevis* attenuated various abnormalities associated with metabolic dysfunction, causing a reduction in the accumulation of mesenteric adipose tissue, increased insulin secretion following glucose challenge, improved plasma cholesterol clearance, and reduced despair-like behavior and basal corticosterone production during FST. This exploratory study suggested that increased microbial GABA production could affect both host metabolism and behavior. Another study found that *A. muciniphila* subtype improves olanzapine-induced glucose homeostasis in mice by downregulating G6Pase and phosphoenolpyruvate carboxykinase overexpression, attenuating insulin resistance, and reducing systemic inflammation by restoring intestinal barrier function (Corb Aron et al., 2021). In addition, one recent ongoing RCT conducted by Gawlik-Kotelnicka et al. found that probiotic administration (including *L. casei*, *L. acidophilus*, and *B. bifidum*) in patients with depression for eight weeks had beneficial effects on the Beck Depression Inventory score, insulin, hs-CRP, and glutathione concentrations (Gawlik-Kotelnicka et al., 2021). They also evaluated the influence of supplementation with a probiotic mixture including *B. longum* Rosell^®^-175 and *L. helveticus* Rosell^®^-52 on depressive symptoms, QoL, inflammation, oxidative stress indices, and fecal microbiota in patients with depression, depending on the metabolic syndrome comorbidity (Gawlik-Kotelnicka et al., 2021). If successful, the trial will establish an easy-to-use and safe treatment option (probiotic supplement) as an adjunctive therapy in patients who are only partially responsive to pharmacological treatment. In a 2-month clinical trial, treatment with a synbiotic formula of *L. acidophilus* PBS066, *L. plantarum* PBS067, and *L. reuteri* PBS072 with active prebiotics could decrease the prevalence of metabolic syndrome and several cardiovascular risk factors and markers of insulin resistance in older patients, which improved their QoL (Cicero et al., 2021). The above-mentioned evidence suggests that some specific probiotics could be used as adjunctive treatment options to treat the comorbidity of depression and metabolic syndrome. Thus, future probiotic intervention studies in large cohorts of patients with comorbid depression and metabolic syndrome and more rigorous RCTs are needed to determine whether probiotics can provide benefits for comorbidity treatment in clinical practice.

## 6 Summary

Accumulating evidence has identified that the gut microbiota actively participates in bidirectional gut-brain communication, which is considered the “second brain” of the human body. The gut microbiota has significant impact on the immune system, brain development, and behavior, and its alterations lead to the onset and progression of several neuropsychiatric disorders, including depression. The exact mechanisms by which the gut microbiota causes or alters depression are not fully understood, although current evidence demonstrates that the gut microbiota can affect the development of depression, mainly through the HPA axis, and inflammation, and modify the level of BDNF. Based on the present scientific discoveries, the gut microbiota may be a novel therapeutic target for the prevention and treatment of depression, with pre-clinical and clinical studies suggesting that several strains of probiotics can provide critical benefits for preventing and treating depression. Due to the lack of direct clinical evidence, it still cannot be recommended that probiotics can replace antidepressant medications as the primary treatment for patients with depression. In addition, depression often coexists with IBS, IBD, and metabolic syndromes, further increasing the risk of mortality. The pathophysiological overlap between depression and its comorbidity makes it feasible to treat these diseases with specific probiotics, which can improve both depressive symptoms and comorbid abnormalities. However, it is noteworthy that these benefits are strain-specific, while other influencing factors, including the type of intervention (add-on versus standalone), intervention content (strain combinations and dosing), delivery modes (tablets, capsules, powders, and freeze-dried formulations), patient population, and disease severity threshold for inclusion should also be considered. There is an urgent need to identify safe and effective novel probiotic strains to prevent and treat depression and its comorbidities. In the future, more rigorous RCTs in larger samples of patients diagnosed with depression with/without comorbidity should explore the optimal probiotic supplement content and dosage, long-term safety, and efficacy, along with an appropriate follow-up to assess relapse rates. Probiotic supplementation may serve as a simple and effective dietary intervention to promote mental well-being among patients with depression and depressed comorbidities.

## Author contributions

ZL, and WX conceived and designed the review. JG, LZ, YC, ZL, and WX analyzed the data. JG, LZ, YC, WL, YW, XL, NZ, LS, XC, YS, ZL, and WX discussed the contents, wrote, reviewed, and edited the manuscript. All authors contributed to the article and approved the submitted version.
